# Bioinorganic Preparation of Hydroxyapatite and Rare Earth Substituted Hydroxyapatite for Biomaterials Applications

**DOI:** 10.1155/2023/7856300

**Published:** 2023-01-23

**Authors:** Suha Q. Al-Shahrabalee, Hussein Alaa Jaber

**Affiliations:** Department of Materials Engineering, University of Technology, Baghdad, Iraq

## Abstract

Rare Earth elements in the lanthanide series are regarded as one of the finest options for the cationic substitution of calcium ions in hydroxyapatite (HA) because of their favorable impact on the biological characteristics of substituted HA. Neodymium and cerium were used to substitute 5% of calcium ions in HA, prepared via the wet precipitation method. Characterization tests for pure and substituted HA were conducted using XRD, FTIR, EDS, and FESEM. The results showed that changing part from calcium ions in hydroxyapatite to Nd and Ce ions altered its structure, composition, and morphology. Regarding the biological tests, the cytotoxicity test revealed a change in IC_50_ for both normal and cancer cell lines, where substitution part of the Ca ions with rare Earth elements led to increasing antitumor activity in comparison with HA without substitution; in addition, antibacterial and fungicide activity was evident for both HA and Nd-Ce/HA, with a modest increase in antibacterial activity of Nd-Ce/HA against *S. epidermidis* and *E. coli* in comparison with HA. These findings may shed light on the process by which Nd and Ce ions improve the biological characteristics of pure HA and the increased potential of these bioceramics.

## 1. Introduction

The development of innovative biomaterials to fix and heal damaged or broken bones is a result of the ongoing demand to improve living quality. The focus at the moment is on bioactive bone repair materials that can speed up the mending process [1]. Bioactive compounds are designed to cause hydroxyapatite (HA) precipitation when in contact with human fluids such as bone regeneration materials, where HA has good properties [2] because it is regarded as one of the essential components of bone and teeth in addition to its capacity to adhere to bone [3]. Their bone-bonding technique is based on the development of a hydroxycarbonate apatite surface layer that corresponds to the crystallographic characteristics and chemical makeup of bone, which is composed primarily of hydroxyapatite (approximately two-thirds) [4, 5]. Substituted hydroxyapatite is gaining popularity because the normal characteristics of hydroxyapatite can be adjusted to accomplish particular biological responses in these biomaterials [6, 7], where even a small amount of doping in hydroxyapatite can cause significant changes in essential characteristics [8–10]. The total energy of the system is increased, and the binding energies between the system and the apatite elements are decreased when one element is replaced by one of the elements that make up HA [11, 12]. Numerous studies have already been conducted on the HA defect structures substituted by cationic ions of various electronic valences. Extensive research attempts to depict the defect structure and the defect formation process. Foreign elements with varying sizes, electronegativity, and valence are probable to make HA unstable after sintering, inducing point defects, and lattice distortions. This will result in the dissolution of the constituent elements in vivo, which in turn leads to the creation of low crystalline hydroxyl carbonate apatite, which will improve the capacity of osseo-integration [13]. Ion substitution is hypothesized to affect the mineralization and remineralization of calcified tissues, as well as the physiological, physical, and chemical features of solid bones and teeth [14]. Today, rare Earth elements are used in industries besides technology and agriculture, such as medicine. Because of their numerous pharmacological and biological characteristics that are helpful in therapeutic settings, lanthanides have gained a lot of interest in the field of medicine. They have been demonstrated to have anticoagulant and antibacterial properties in addition to phosphate-binding properties, with the majority of their effects attributed to their impact on Ca^2+^-dependent activities, which may be included in specific diseases [15]. Biological aspects of lanthanide ions reveal several key characteristics where incorporation of elements from lanthanide series in biomaterials can lead to improving various properties such as biocompatibility and anticancer effect [16, 17]. Numerous investigations showed that the accumulation of rare Earth elements in animals' bones, muscles, kidneys, or liver was unaffected by the long-term administration of orally replaced rare Earth elements [18]. Neodymium (Nd) is a light rare Earth element that is employed in biomaterials and hardly exhibits cytotoxicity [19]. It is used in the health sector and may be found in various medical devices [20]. Along with neodymium, cerium (Ce) is a rare element that is utilized in medicine. It has been shown that cerium has cytotoxic effects on cancer cells and is capable of slowing the growth of a variety of bacteria. Additionally, it has an impact on metabolism, where the amount of cerium that is absorbed may have an impact on other cellular processes like immune system support or cell proliferation [21]. The antibacterial effectiveness rose as the amount of Ce increased, proving that cerium replaced calcium and improved the antibacterial function. It was demonstrated that the nanoparticles of Ce/HA produced by a special sol-gel technique possess antibacterial properties in both static and dynamic interaction with the bacteria [22], where the antibacterial capabilities against *Escherichia coli* and *Staphylococcus aureus* bacteria increased as cerium content increased, with *E. coli* being more effective [23].

In order to generate a new biomaterial with unique qualities, this study sought to combine the biological advantages of neodymium and cerium. This investigation looked into how the partial replacement of Nd and Ce ions for Ca ions influenced the structure and biological characteristics of HA made utilizing the wet precipitation method.

## 2. Materials and Methods

### 2.1. Materials

The materials employed in this study were (NH_4_)_2_HPO_4_, Ca(NO_3_)_2_.4H_2_O, Nd(NO_3_)_3_.6H_2_O, Ce(NO_3_)_3_.6H_2_O, and NH_4_OH which were supplied by Merck, Himedia, Sigma Aldrich, Avonchem, Chem-Lab, respectively, with a purity of 99.9% for all used salts and a purity of 25% for ammonia solution.

### 2.2. Synthesis of HA and Nd-Ce/HA

**Figure 1:**
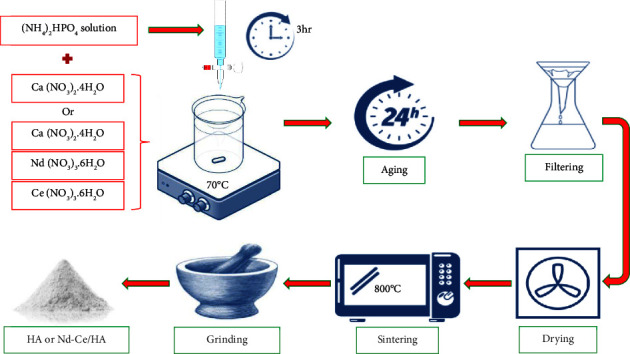
Preparation steps of HA and Nd-Ce/HA.

Wet chemical precipitation is a method of producing hydroxyapatite that involves using aqueous solutions containing Ca and phosphorus ions which chemically react at a controlled temperature and pH to produce HA with properties similar to bone and dental tissue. The technique shown in Figure 1 was used to create nano-HA and Nd-Ce/substituted hydroxyapatite (Nd-Ce/HA) with 5% ionic substitution (2.5% Nd and 2.5% Ce) by performing the following steps:

1. Prepared a 0.1 M solution of di-ammonium hydrogen phosphate, and used ammonia to adjust the pH to 10.5 while stirring the solution for about 30 minutes
2. Prepared 0.167 M of a solution containing nitrate salts by mixing calcium nitrate salt with deionized water during the synthesis of hydroxyapatite and mixing calcium nitrate, neodymium nitrate, and cerium nitrate with deionized water during the production of Nd-Ce/HA
3. For more than three hours at 70°C, added dropwise solutions of di-ammonium hydrogen phosphate were to solutions of nitrate salts
4. After complete dropping, heating was stopped, and NH_4_OH was progressively added to raise the pH of the final solution to 11
5. Age the solution on a stirrer for one day (at room temperature)
6. After aging, the mixture stir was stopped and left for long enough to allow the hydroxyapatite to precipitate and separate it from the liquid portion
7. The liquid portion of the mixture was discarded and saved the precipitate, then washed with deionized water, and filtered using filter paper
8. A sufficient amount of time was used to dry the precipitate at 100°C, sintered at 800°C, and then ground the final prepared materials

## 3. Specimens Characterization and Biological Tests

### 3.1. X-Ray Diffraction Technology (XRD)

Following the completion of the wet precipitation procedure, the synthesis of hydroxyapatite and Nd-Ce/HA was confirmed using XRD-Philips X'pert PRO, Holland operating with CuK*α* radiation (*λ* = 1.5405 Å). The data were drawn through a step of 0.05° point/second in a 2*θ* range of 10°–80°. With the aid of the software X'pert highscore plus, the collected spectra were examined.

### 3.2. Fourier Transform Infrared Spectroscopy (FTIR)

The chemical groups of HA and substituted HA were identified using an FTIR test on Bruker Tensor 27 IR, Germany. Where the transmittance mode was adopted, the acquired FTIR spectra will be representative of the entire sample.

### 3.3. Field Emission Scanning Electron Microscope (FESEM) and Energy Dispersive Spectroscopy (EDS)

FESEM and EDS with mapping were done by using Zeiss Sigma 300-HV, Germany, where field emission scanning electron microscopy (FESEM) was used to observe the microstructure of HA and Nd-Ce/HA powder specimens, whereas EDS with mapping was employed to notify the chemical composition of the produced materials and determine the elemental distribution of the elements inside the powders.

### 3.4. Antibacterial and Fungicide Activity

Human in our daily life is exposed to many types of bacteria and fungi; therefore, the synthesis of a new biomaterial must be resistant to these microorganisms. The microorganisms that were taken into account in this study were several kinds of Gram-negative and Gram-positive bacteria, such as *Escherichia Coli* (generally found in the human digestive tract flora) [24], *Staphylococcus aureus* (the pathogen with the most epidemiological evidence) [25], *Streptococcus mutans* (frequently identified in the oral cavity and cardiovascular) [26], *Staphylococcus epidermidis* (natural inhabitant of the mucosa and the skin) [27, 28], and finally, a type of fungi which is *Candida albicans* (a component of the natural microbiota of the skin) [29]. Activation of microorganisms was done in the lab according to the standard conditions, and the examination of biological activity was done according to the method of well diffusion [30]. In the case of antibacterial activity, gentamicin tablets containing ten micrograms concentrations were used as a comparison, whereas nystatin was used as a comparison for fungicide activity.

### 3.5. Cytotoxicity Assay

This in vitro test was carried out to investigate the possible cytotoxicity of HA and Nd-Ce/HA on WRL68 and MG63 cell lines. The WRL68 human hepatic cell line's morphology is similar to that of hepatocytes and primary cultures. It has been demonstrated that cells release alpha-fetoprotein and albumin as well as liver-specific enzymes like alanine aminotransferase [31], whereas osteosarcomas (MG63) are cancerous bone tumors made up of cells with atypical cellular processes. Malignant bone tumors give rise to osteosarcoma cells. Although these cells exhibit certain osteoblastic characteristics, their chromosomal abnormalities result in aberrant molecular and cellular processes [32].

The experiments were done in triplicate, and the IC_50_values (half-maximal inhibitory concentration) of the samples were determined by using the curve of log dosage inhibition. In 100 *µ*l of Roswell Park Memorial Institute (RPMI-1640), osteosarcoma cells (MG63) were grown. The medium contained 10% fetal bovine serum (FBS) (institute medium). Regarding cell attachment, MG63 cells were incubated overnight at a temperature of 37°C with 5% CO_2_.

The cells (from 1 × 10^4^ to 1 × 10^6^ cells mL^−1^) were cultured in well plates (96) to a final volume of 200 *µ*L/well. These plates were coated with sterilized parafilm, stirred, and then incubated for 24 hrs at 37°C in the presence of 5% CO_2_. The medium was taken after incubation, and 200 *µ*L of serial dilution of hydroxyapatite and Nd-Ce/HA (6.25, 12.5, 25, 50, 100, 200, and 400 *µ*g/mL) was added to the plate's wells. Each concentration and control was tested in triplicate. At 37°C and 5% CO_2_, the plates were incubated for 24 hrs, and then, 10 *µ*L of MTT solution was added to each well after exposure to HA and Nd-Ce/HA. The plates were then incubated for 4 hours at 37°C with 5% CO_2_. After carefully removing the media, each well was filled with 100 *µ*L of the dissolving solution and incubated for 30 min. At a wavelength of 575 nm, the absorbance was measured using an ELISA reader (Bio-rad, Germany). The viability was calculated using statistical analysis of the optical density values, according to the following equation:

(1) $$\begin{array}{c} Viability\left(\%\right)=\left(\frac{optical\;density\;of\;sample}{optical\;density\;of\;control}\right)\times 100\text{.} \end{array}$$

## 4. Results and Discussions

### 4.1. X-Ray Diffraction Technology

**Figure 2:**
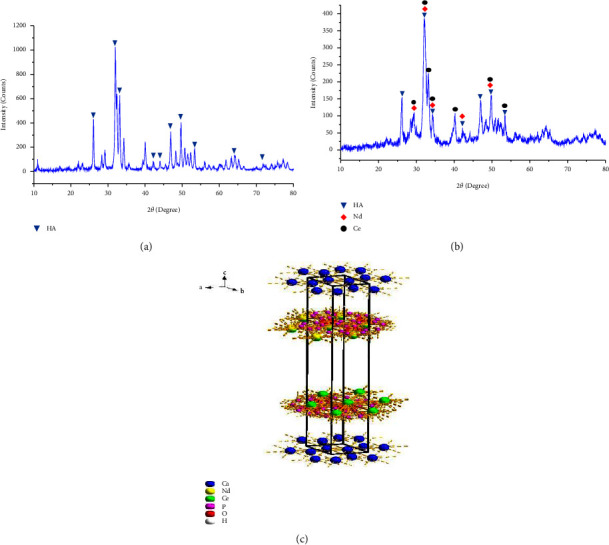
XRD test of (a) prepared hydroxyapatite, (b) Nd-Ce/HA, and (c) crystal structure of Nd-Ce/HA.

The crystalline nature of hydroxyapatite was obvious from the pattern of XRD illustrated in Figure 2(a). Peaks with high intensity presented at 2*θ* values 26.098°, 31.978°, 34.284°, 42.276°, 43.996°, 46.934°, 49.643°, 53.322°, 64.234°, and 72.117° are belonged to hydroxyapatite according to JCPDS cards number 00-001-1008 and 96-900-1234.

In the case of the substituted hydroxyapatite (Figure 2(b)), the X-ray examination exhibited peaks belonging to HA, Nd, and Ce; the peaks belonging to the formation of hydroxyapatite appeared clearly at 2*θ* value 26.189°, 32.030°, 34.344°, 42.336°, 47.058°, 49.809°, and 53.440° according to JCPDS cards number 00-001-1008 and 96-900-1234, while the peaks relate to the presence of neodymium ions inside the structure of hydroxyapatite were 29.299°, 32.030°, 34.344°, 42.336°, and 49.809° according to JCPDS cards number 00-039-0914 and 01-089-2922. Finally, the peaks belonging to Ce were 29.299°, 32.030°, 33.219°, 34.344°, 40.214°, 49.809°, and 53.440° due to the presence of cerium ions within the hydroxyapatite structure corresponding to JCPDS cards number 01-076-2371, 01-076-2434, 01-078-0640, and 01-089-2728.

The crystal system of hydroxyapatite is hexagonal, where the lattice constant *a* = *b* ≠ *c*, but the value of these constants may change after the substitution process, and thus, it is necessary to identify some parameters (Table 1) that help us to compare these constants before and after cationic substitution. *d*-spacing (interplaner spacing) was calculated by applying Bragg's low:

(2) $$\begin{array}{c} n\lambda =2d\;\sin\;\theta \text{,} \end{array}$$

where *n* = 1 (order of diffraction), *λ* = 1.5405 Å (wavelength of incident X-ray), *d* = interplaner spacing (Å), *θ* = peak position (Radians), and the lattice parameter values were calculated by applying the following equation:

(3) $$\begin{array}{c} \frac{1}{d^{2}}=\frac{4}{3}\left[\frac{h^{2}+k^{2}+hk}{a^{2}}\right]+\frac{l^{2}}{c^{2}}\text{,} \end{array}$$

where *d* = interplaner spacing (Å); *h*, *k*, and *l* = Miller indices; *a*, *b*, and *c* = lattice parameter.

After performing the necessary arithmetic operations to calculate the lattice constants, it became clear that the values of the constants *a* and *b* before the operation of ionic substitution had slightly changed compared to them after the substitution, where *a* and *b* were 9.445 Å for HA, and they became equal to 9.343 Å after substitution with neodymium and cerium ions. As for the value of the constant *c*, it was almost equal before and after the substitution process.

The ionic substitution in the hydroxyapatite lattice causes the broadening of the peaks brought on by a reduction in crystallite size and an increase in lattice disorder as it is evident from Table 2 that Nd-Ce/HA exhibits more lattice strain. The crystallite sizes calculated using the Scherrer equation can also be used to categorize the hydroxyapatite samples as nano-HA. The results show that the metals' insertion into the HA lattice increased the specific surface area (*S*_BET_), which is connected to the smaller crystallite sizes of these samples [33]. Adjusting the crystallinity level promotes cell adhesion and migration, speeds up wound healing, and has varying biodegradation rates [34].

Lanthanide ions are of tremendous interest for incorporation into HA in biological applications because of their remarkable affinity for Ca^2+^ sites. An ion-exchange mechanism for trivalent lanthanides specifically explains this strong affinity; the binding constant for the exchange increases with decreasing ion size. The ion exchangeability is significantly influenced by the charge density fluctuation caused by the lattice parameters adaption when trivalent cations replace Ca^2+^ ions. The charge imbalance is balanced out either by creating unoccupied cation sites or by losing a proton from hydroxide. Doped HA's demonstrated a general decrease in crystallinity and an increase in surface area when compared to the pure phase after being substituted with rare Earth elements. For use in biological fluorescent labeling, lanthanide or actinides-HA composites showed remarkable luminescence qualities and show promise (for example, in magnetic resonance imaging and multi-imaging diagnostic on single photon emission computed tomography) [35].

As shown in Figure 2(c), the structure of Nd-Ce/HA was created utilizing diamond software in accordance with the results of an XRD test in order to facilitate further investigation. It is evident that Ca ions are arranged at the top and bottom of the structure, and they are in direct contact with hydrogen ions at the original coordination, while the neodymium and cerium ions were associated with the phosphorous and oxygen ions in the form of two opposite networks in the middle of the structure, which in turn were associated with the calcium ions.

### 4.2. Fourier Transform Infrared Spectroscopy

**Figure 3:**
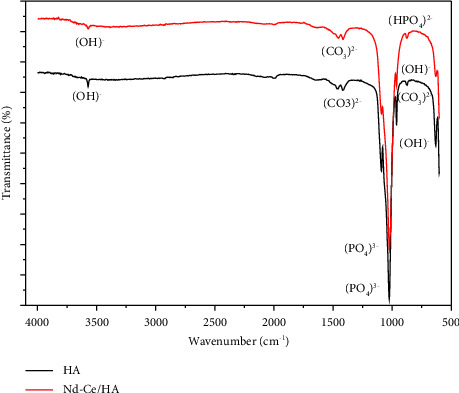
FTIR test of prepared HA and Nd-Ce/HA.

Figure 3 illustrates the FTIR spectra, which show the primary functional groups of HA and substituted HA. The vibrational mode of the hydroxyl (OH)^−^ group can be recognized at the bands of 3571.98 cm^−1^ as well as 631.16 cm^−1^ for HA and 3570.88 cm^−1^ in addition to 629.96 cm^−1^ for Nd-Ce/HA. On the other hand, the presence of the (HPO_4_)^2−^ group at the band 874.44 cm^−1^ for Nd-Ce/HA indicates calcium deficiency, which is one of the substituted HA characteristics. (PO_4_)^3−^ is another essential functional group in HA, which can be observed at 1090.47 cm^−1^, 1024.22 cm^−1^, and 962.94 cm^−1^ for HA compared with peaks of 1090.30 cm^−1^, 1022.77 cm^−1^, and 962.95 cm^−1^ for Nd-Ce/HA. The substitution of 5% of calcium ions with Nd and Ce led to a decrease in the transmittance of the O-P-O stretching mode and broadening in the band of the PO_4_ group. In spite of that, there was an observed change in transmittance and wavenumber of the HA groups after substitution as a consequence of Nd-Ce electrostatic interactions with (PO_4_)^3−^ [36]. During the aging process, which takes 24 hrs, HA and Nd-Ce/HA were subjected to carbon in the atmosphere, and because of that, (CO_3_)^2−^ group appeared in bands of HA (873.97 cm^−1^, 1418.28 cm^−1^, and 1465.89 cm^−1^) and substituted HA (1416.66 cm^−1^ and 1455.36 cm^−1^). However, because the bone mineral phase is carbonated, their presence may promote the bioactivity of HA rather than being a cause for worry [37].

### 4.3. Microstructure and Chemical Composition Investigation by FESEM and EDS Mapping

**Figure 4:**
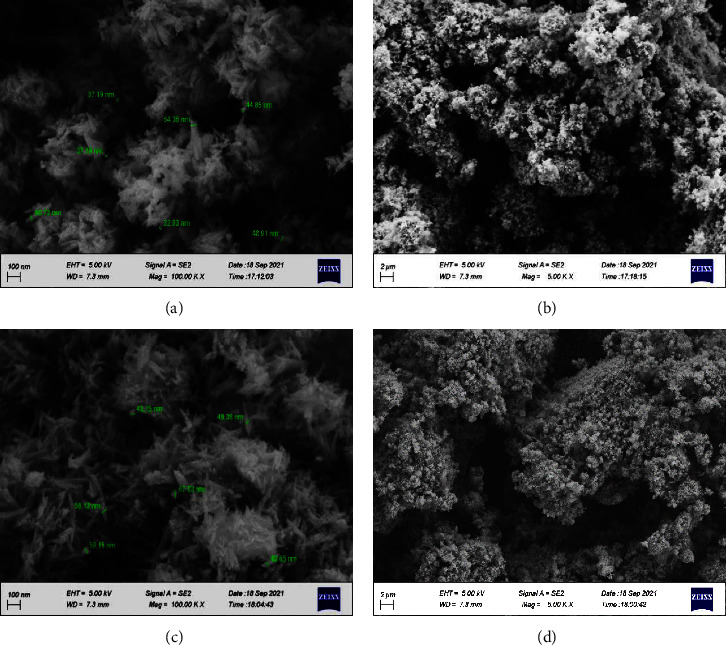
Images of FESEM (a) HA with particle size, (b) HA, (c) Nd-Ce/HA with particle size, and (d) Nd-Ce/HA.

Figures 4(a) and 4(b) show the FESEM image of nanohydroxyapatite powder produced by the wet precipitation technique, which was analyzed at a resolution of 100 nm and 2 *µ*m with the magnification of 100.00 KX and 5.00 KX, respectively. At a magnification of 100.00 KX, HA appears in an elongated form with a relatively uniform distribution, where the morphology of HA has been affected by the temperature of the reaction (70°C) and pH of the synthesized solution, which was a highly alkaline medium. The presence of neodymium and cerium in the hydroxyapatite structure results in the formation of new morphologies within it as shown in Figures 4(c) and 4(d). The rod-like structure belongs to neodymium, and the feather-like structure belongs to cerium, in addition to the elongated particles of hydroxyapatite; this was obvious from the FESEM image with a magnification of 100.00 KX and resolution of 100 nm. From these images, it is evident that the final structure of hydroxyapatite depends on the conditions of preparation and elements added during the synthesis process [38–40].

From Figures 4(a)–4(c), it is clear that both materials have a nanosize, and one of the potential mechanisms for improved tissue growth and cell adhesion is seen with nanostructured biomaterials, where the greater levels of particular protein interactions are encouraged by nanostructured materials, which also more effectively drive the production of new bone [34]. Also, the osteoinductive properties improve after the incorporation of trace elements in the HA structure [41].

**Figure 5:**
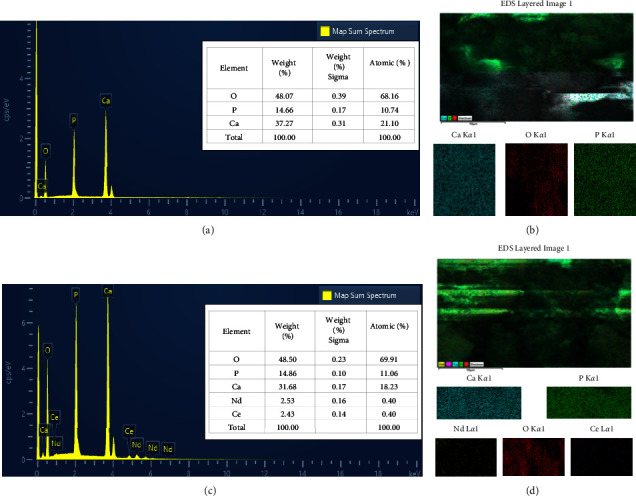
Chemical composition with elemental distribution to illustrate (a) EDS of HA, (b) HA mapping, (c) EDS of Nd-Ce/HA, and (d) Nd-Ce/HA mapping.

The chemical composition of HA and Nd-Ce/HA was confirmed by EDS analysis. The essential elements that HA was composed of, such as Ca, P, and O, were visible in Figure 5(a), with their elemental distribution by mapping in Figure 5(b). On the other hand, the EDS analysis of Nd-Ce/HA revealed the presence of Nd and Ce, as well as Ca, P, and O, as shown in Figure 5(c), with their mapping in Figure 5(d). Atomic percentage in the EDS test shows that the Ca/P ratio is 1.9 for HA, while after the substitution, the ratio of Ca + Nd + Ce/P was 1.7, which is very close to the Ca/P ratio of human bone.

### 4.4. Test of Antibacterial and Fungicide Activity

**Figure 6:**
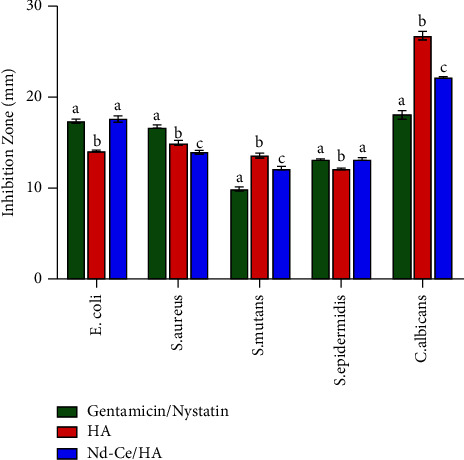
Bar graph exhibited antibacterial and fungicide activity of HA and Nd-Ce/HA (different letters (*a*, *b*, *c*) are significant at *p* < 0.05).

Both HA and Nd-Ce/HA showed an inhibition zone against all kinds of microorganisms used in this test. The presence of neodymium and cerium in HA exhibited antibacterial and fungicide activity as the prepared hydroxyapatite without substitution is obvious in Figure 6 and Table 3. The inhibition zone was almost close for both HA and Nd-Ce/HA concerning *S. aureus*, *S. mutans*, and *S. epidermidis*. As for *E. coli*, Nd-Ce/HA has a bigger inhibition zone compared to HA, which exhibited a bigger inhibition zone for fungi, and these data were analyzed by 2-way ANOVA (Tukey test) to compare them as is clear in Table 4. Standard treatments for bone infections remain ineffective, even with extended high-dose regimens. As a result, bone-targeting medication delivery devices have been developed. Hydroxyapatite is a common substance used as a system matrix because of its porosity where it has the potential to offer adequate loading and long-term release of antibacterial chemicals, which is critical for such a system's antibacterial efficiency [42]. Commercial HA had no effect on Gram-positive or Gram-negative bacteria. Surprisingly, the manner of hydroxyapatite preparation plays a considerable impact in determining its efficacy against microorganisms; for instance, HA samples prepared by microwave-assisted combustion method had a superior resistance against Gram-positive and Gram-negative bacteria. Nanoparticles can cause antibacterial effects through various processes, including changes to the cell wall and cytoplasm, membrane changes, and respiratory inhibition [43]. Thus, from the obtained results, the wet precipitation method can also produce HA with antibacterial properties. The presence of neodymium and cerium in HA exhibited resistance to bacteria and fungi because of their characteristics of resistance to the microorganism [35]. The activity is linked to the Nd ratio, which resulted in an increase in the surface-to-volume ratio of nanoparticles, resulting in better interaction with microorganisms [44]. Moreover, the presence of cerium results in bacterial [45] and fungicide activity [46] because Ce increases antibacterial action, pathogen inhibition, and regeneration [35].

### 4.5. Cytotoxicity Assay

**Figure 7:**
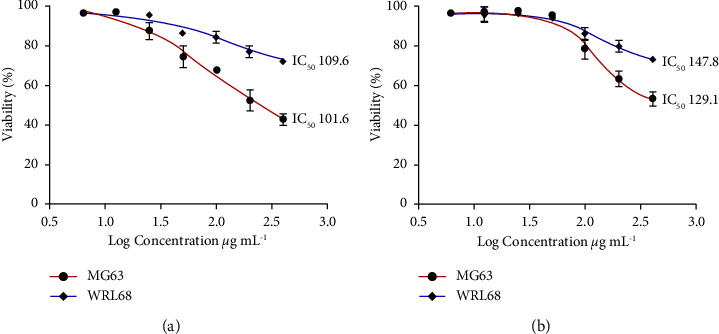
Cell survival curve (mean ± SD%) of MG63 and WRL68 after treatment with (a) HA and (b) Nd-Ce/HA for 24 hrs., with the details of cytotoxicity effect.

As it was evident in Figure 7(a), HA exhibited significant antitumor activity (*p* < 0.0001) against MG63 cells in a dose-dependent pattern, and the viability was 85.30 ± 1.75, 75.54 ± 5.21, 66.05 ± 2.70, 51.85 ± 3.61, and 42.79 ± 2.79% for the concentrations 25, 50, 100, 200, and 400 *μ*g/mL, respectively. By comparing the results with WRL68 in Table 5, only HA treatment at concentrations 6.25 and 12.5 *μ*g/mL exhibited no significant differences in MG63 cell inhibition rate with a calculated IC_50_ of 101.6 *μ*g/mL and 109.6 *μ*g/mL for MG63 and WRL68, respectively. On the other hand, Nd-Ce/HA also showed significant antitumor activity (*p* < 0.0001) against MG63 cells. Where the results in Figure 7(b) showed that there was no difference between normal and cancer cells at low concentrations (6.25, 12.50, 25, 50) *μ*g/mL, there were active results on the cancer cell line when the concentration doubled to 200 *μ*g/mL and 400 *μ*g/mL with a reduction rate of 35.72 ± 3.59% and 46.6 ± 3.31%, respectively, and in addition to that, the calculated IC_50_ for Nd-Ce/HA was 129.1 *μ*g/mL and 147.8 *μ*g/mL for MG63 and WRL68, respectively. Ce and Nd have anticancer properties because of their cytotoxic agents; the increased cytotoxicity of these ions is accompanied by antioxidants, and the Ce compound's activity in addition to that of the Nd compound may be attributed to the coordinative bonds [47]. It is clear that the presence of neodymium and cerium in the crystalline structure of hydroxyapatite led to an evident change in the resistance of the material to cancer cells compared to the material (pure HA) that does not contain rare Earth elements [48].

## 5. Conclusion

The incorporation of rare Earth elements (Nd and Ce) inside the structure of hydroxyapatite leads to a change in the morphology, composition, and biological properties of hydroxyapatite. Using rare Earth elements such as neodymium and cerium to substitute part of calcium ions by wet precipitation method leads to adding rod-like and feather-like shapes to the morphology of HA and changing the final chemical composition of the prepared powder. Regarding the biological properties, both HA and Nd-Ce/HA exhibited antibacterial and fungicide activity as one of the inherent properties of the prepared HA. Finally, the cytotoxicity test showed an evident change in the influence of Nd-Ce/HA on the WRL68 and MG63 cell lines compared with the effect of hydroxyapatite before substitution.

## Figures and Tables

**Table 1:** Essential XRD data of HA and Nd-Ce/HA.

Peak position 2*θ* (°)		FWHM		Interplaner spacing *d* (Å)		Miller indices (hkl)	
HA	Nd-Ce/HA	HA	Nd-Ce/HA	HA	Nd-Ce/HA	HA	Nd-Ce/HA
26.098	26.189	0.1968	0.2952	3.41449	3.40286	002	002
31.9779	32.030	0.1968	0.3936	2.79881	2.79437	121	112
34.2839	33.219	0.246	0.3936	2.61565	2.69703	022	030
42.2759	34.344	0.3936	0.3936	2.13784	2.61119	032	022
43.9959	42.336	0.1968	0.7872	2.05817	2.13495	113	032
46.9342	47.058	0.3936	0.2952	1.93595	1.93114	222	222
49.6435	49.809	0.2952	0.2952	1.83645	1.83073	123	123
53.3215	53.440	0.2952	0.48	1.71813	1.71317	004	004
64.2344	—	0.3936	—	1.45008	—	233	—
72.1167	—	0.7872	—	1.30976	—	250	—

**Table 2:** Important factors calculated from the extracted data of the XRD test.

Prepared material	Lattice parameter			Crystallite size (nm)	Lattice strain
	*a* (Å)	*b* (Å)	*c* (Å)		
HA	9.4448	9.4448	6.8290	43.88	0.0030
Nd-Ce/HA	9.3428	9.3428	6.8057	21.84	0.0064

**Table 3:** Inhibition zone (mean ± standard deviation) of gentamicin/nystatin, HA, and Nd-Ce/HA.

Type of microorganism	Gentamicin/nystatin		HA		Nd-Ce/HA	
	Mean ± SD (mm)	Sample number	Mean ± SD (mm)	Sample number	Mean ± SD (mm)	Sample number
*E. coli*	17.37 ± 0.23	3	13.97 ± 0.15	3	17.60 ± 0.38	3
*S. aureus*	16.60 ± 0.31	3	14.90 ± 0.26	3	13.97 ± 0.15	3
*S. mutans*	9.90 ± 0.21	3	13.53 ± 0.27	3	12.13 ± 0.19	3
*S. epidermidis*	13.10 ± 0.06	3	12.13 ± 0.09	3	13.17 ± 0.17	3
*C. albicans*	18.07 ± 0.46	3	26.77 ± 0.46	3	22.17 ± 0.12	3

**Table 4:** Tukey's multiple comparisons tests between gentamicin/nystatin, HA, and Nd-Ce/HA (NS means the probability (*p*) is nonsignificant, ^*∗*^ means *p* < 0.05, and ^*∗∗*^ means *p* < 0.01).

Type of sample	Summary	Adjusted *p*-value
*E. coli*		
Gentamicin vs. HA	^*∗∗*^	<0.0001
Gentamicin vs. Nd-Ce/HA	NS	0.8064
HA vs. Nd-Ce/HA	^*∗∗*^	<0.0001

*S. aureus*		
Gentamicin vs. HA	^*∗∗*^	0.0002
Gentamicin vs. Nd-Ce/HA	^*∗∗*^	<0.0001
HA vs. Nd-Ce/HA	^*∗*^	0.0454

*S. mutans*		
Gentamicin vs. HA	^*∗∗*^	<0.0001
Gentamicin vs. Nd-Ce/HA	^*∗∗*^	<0.0001
HA vs. Nd-Ce/HA	^*∗∗*^	0.0020

*S. epidermidis*		
Gentamicin vs. HA	^*∗*^	0.0372
Gentamicin vs. Nd-Ce/HA	NS	0.9825
HA vs. Nd-Ce/HA	^*∗*^	0.0246

*C. albicans*		
Nystatin vs. HA	^*∗∗*^	<0.0001
Nystatin vs. Nd-Ce/HA	^*∗∗*^	<0.0001
HA vs. Nd-Ce/HA	^*∗∗*^	<0.0001

**Table 5:** Comparison between the cytotoxicity effect of HA and Nd-Ce/HA on cell lines of MG63 and WRL68.

Concentration (*μ*g/mL)	Viability %					
	MG63			WRL68		
	Mean ± SD		Specimens number	Mean ± SD		Specimens number
	HA	Nd-Ce/HA		HA	Nd-Ce/HA	
400	42.79 ± 2.79	53.40 ± 3.31	3	62.27 ± 4.63	72.65 ± 1.96	3
200	51.85 ± 3.61	64.28 ± 3.59	3	73.64 ± 4.65	80.21 ± 3.11	3
100	66.05 ± 2.70	79.50 ± 6.59	3	76.81 ± 3.30	85.65 ± 3.32	3
50	75.54 ± 5.21	95.45 ± 0.88	3	89.64 ± 4.84	94.17 ± 0.77	3
25	85.30 ± 1.75	97.15 ± 1.35	3	95.37 ± 0.90	96.18 ± 0.23	3
12.50	95.33 ± 0.79	95.87 ± 3.28	3	96.64 ± 0.70	94.29 ± 2.98	3
6.25	96.10 ± 1.71	95.99 ± 0.96	3	95.68 ± 0.41	96.07 ± 0.12	3

## Data Availability

All data used to support the findings of this study are included in the article.
